# Saturation and periodic self-stress in geometric auxetics

**DOI:** 10.1098/rsos.220765

**Published:** 2022-08-31

**Authors:** Ciprian S. Borcea, Ileana Streinu

**Affiliations:** ^1^ Department of Mathematics, Rider University, Lawrenceville, NJ, USA; ^2^ Department of Computer Science Smith College, Northampton, MA, USA

**Keywords:** periodic framework, auxetic deformation, self-stress, matroid basis polytope, cognate frameworks

## Abstract

The auxetic structures considered in this paper are three-dimensional periodic bar-and-joint frameworks. We start with the specific purpose of obtaining an auxetic design with underlying periodic graph of low valency. Adapting a general methodology, we produce an initial framework with valency seven and one degree of freedom. Then, we describe a saturation process, whereby edge orbits are added up to valency 16, with no alteration of the deformation path. This is reflected in a large dimension for the space of periodic self-stresses. The saturated version has higher crystallographic symmetry and allows a precise description of the deformation trajectory. Reducing saturation by adequate removal of edge orbits results in vast numbers of distinct auxetic designs which obey the same kinematics.

## 1. Introduction

A periodic bar-and-joint framework can be modified by addition or removal of edges. Obviously, preservation of periodicity requires that such additions or removals be performed on entire orbits of edges, that is, equivalence classes of edges under periodicity. The case of interest for us will be when these operations do not alter locally the kinematics of the structure. Thus, in more technical language, we are concerned with spaces of periodic self-stresses in a local deformation neighbourhood of a given periodic framework [1]. The term saturation will be used for the process of adding orbits of edges without changing the local deformation space.

An important motivation for undertaking this study stems from the role of saturation in *geometric auxetics*. In materials science, auxetic behaviour refers to lateral widening upon stretching and geometric auxetics gives a precise, autonomous mathematical definition to this type of structural deformation [2,3]. Suppose an auxetic framework with one degree of freedom allows saturation; then, by adequate addition and removal of edge orbits, one obtains various distinct, yet kinematically equivalent structures. By analogy with cognate linkages in mechanism design [4], we call them *cognate frameworks*. It will be seen below that a *matroid basis polytope* is controlling the combinatorial possibilities.

Since geometric auxetics works in arbitrary dimension, saturation scenarios can be pursued in any dimension *d* ≥ 2. However, in order to remain specific and streamline the necessary theoretical background from [1–3], we present our main investigation as the unfolding of the following problem in three-dimensional auxetic design: find periodic frameworks which have one degree of freedom, are auxetic and have underlying graphs with low valency at all vertices. When aiming at a low number of vertex orbits as well, it appears that frameworks with four vertex orbits are already apt to illustrate the phenomena of interest. Consequently, our focus will be on frameworks related to a particular initial design with *n* = 4 vertex orbits, *m* = 14 edge orbits and valency seven. Saturation will increase the edge orbits to 32 and valency to 16.

Our study is strictly mathematical and relies on geometric foundations for periodic framework deformations [1], auxetics [2] and auxetic design [3]. Even so, the specific three-dimensional frameworks considered and displayed here can be looked at from the more traditional perspective of materials science. There are many surveys on the appertaining literature [5–14]. In dimension two, the importance of recognizing the same kinematic behaviour for different auxetic models has been pointed out in [15]. Additive manufacturing of metamaterials allows periodic structures with connectivities unrestricted by the proximity constraints of atom-and-bond crystal frameworks and it will be noticed that the long edges in our designs represent middle-range interactions.

The paper is organized as follows. In §2, we describe the framework design process, which illustrates the general principles of [3] but makes several particular and simplifying choices. Section 3 shows that these choices lead to a saturated version with enhanced crystallographic symmetry. The one-dimensional deformation space of the saturated framework is introduced first in a pictorial and intuitive manner in §4 and then precisely described in explicit formulae in §5. The auxetic interval is also determined at this stage. Section 6 examines the presence of framework self-crossings, which is important for applications. Section 7 relies on more detailed computations with the *rigidity matrix* of the saturated framework, which is a 32 × 21 matrix of rank 14. The *matroid basis polytope* associated to this matrix is a combinatorial expression of all the possibilities of eliminating self-stress and reverting to 14 edge orbits. Myriad cognate frameworks result from this process. Section 8 presents, as a companion, a brief planar study of saturation related to more familiar designs. In this case, the matroid basis polytope has 282 vertices (labelling cognate structures) and dimension 11. The conclusion is formulated in §9.

## 2. Framework design

As mentioned, our starting point is the problem of designing auxetic periodic frameworks with one degree of freedom and low valency. The first desideratum is natural in view of the fact that several degrees of freedom would leave open the question about finding and selecting an auxetic trajectory, while the second desideratum is suggested by three-dimensional applications to metamaterial design, where low valency facilitates additive manufacturing [16].

In dimension three, a periodic framework with *n* vertex orbits and one degree of freedom would need, by counting formulae, *m* = 3*n* + 2 edge orbits which impose independent constraints on infinitesimal deformations [1]. Thus, connected frameworks, would have ‘mean valency’ 2*m*/*n* = 6 + (4/*n*) and cannot avoid vertices where the number of incident edges is at least seven. For *n* = 2, one needs valency eight and families of auxetic blueprints can be found in [17], with three-dimensional printed samples and compression experiments described in [18].

The type of framework studied in the present paper was designed with the purpose of reducing the vertex valency to seven, more precisely, the initial underlying 3-periodic graph (G,Γ) has *n* = 4 vertex orbits and *m* = 14 edge orbits, with a quotient multigraph G/Γ represented by a quadrilateral with edge multiplicities (3, 4, 3, 4).

### 2.1. Design of the initial periodic framework

**Figure 1.**
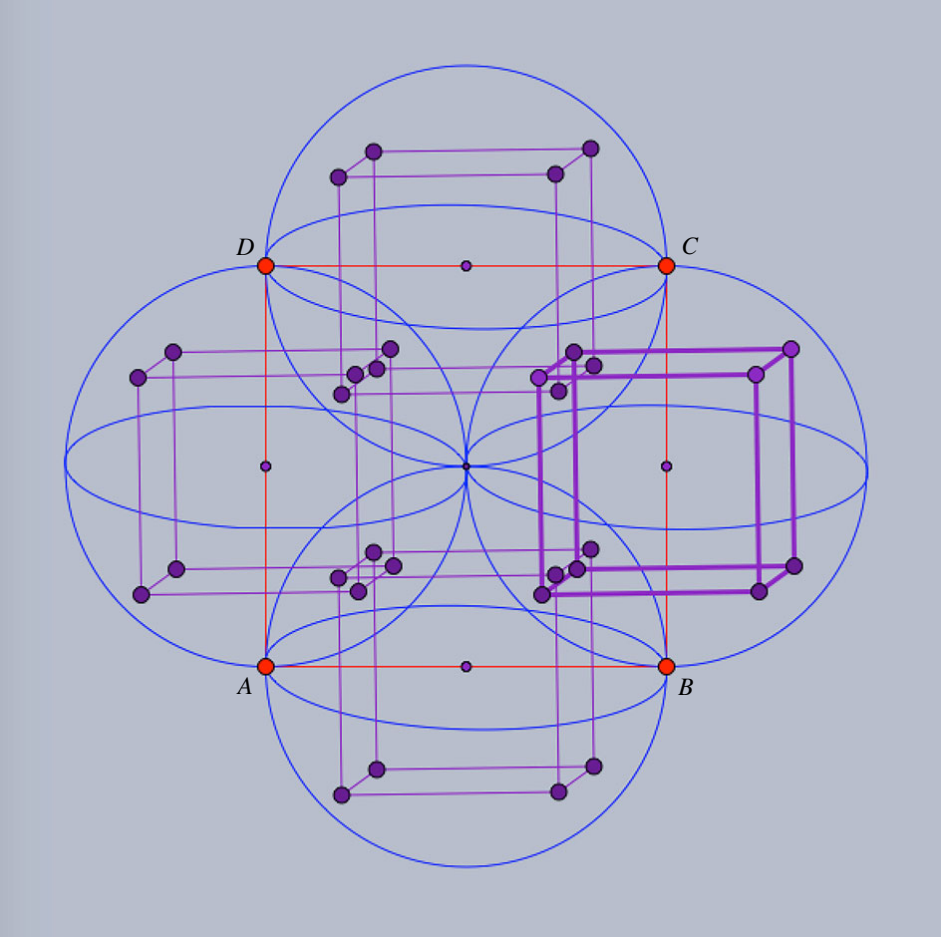
Design setup: the reduced quotient graph is placed as a square *ABCD*; the four congruent spheres with diameters given by the edges *AB*, *BC*, *CD*, *DA* are traced and each sphere contains a translated copy of an inscribed cube with two faces parallel to plane ABCD.

The general auxetic design methodology presented in [3] allows specialized choices for the placement of the reduced quotient graph and the ensuing periodicity lattice. Our reduced quotient graph is a quadrilateral and will be placed as a square *ABCD* in *R*^3^. Aiming at a *cubic periodicity lattice* for the initial framework, we identify by translation the four congruent spheres with diameters *AB*, *BC*, *CD*, *DA* and consider the inscribed cube with two faces parallel to plane *ABCD*, as shown in figure 1. The periodicity lattice Γ will be generated by the edge vectors of this inscribed cube.

**Figure 2.**
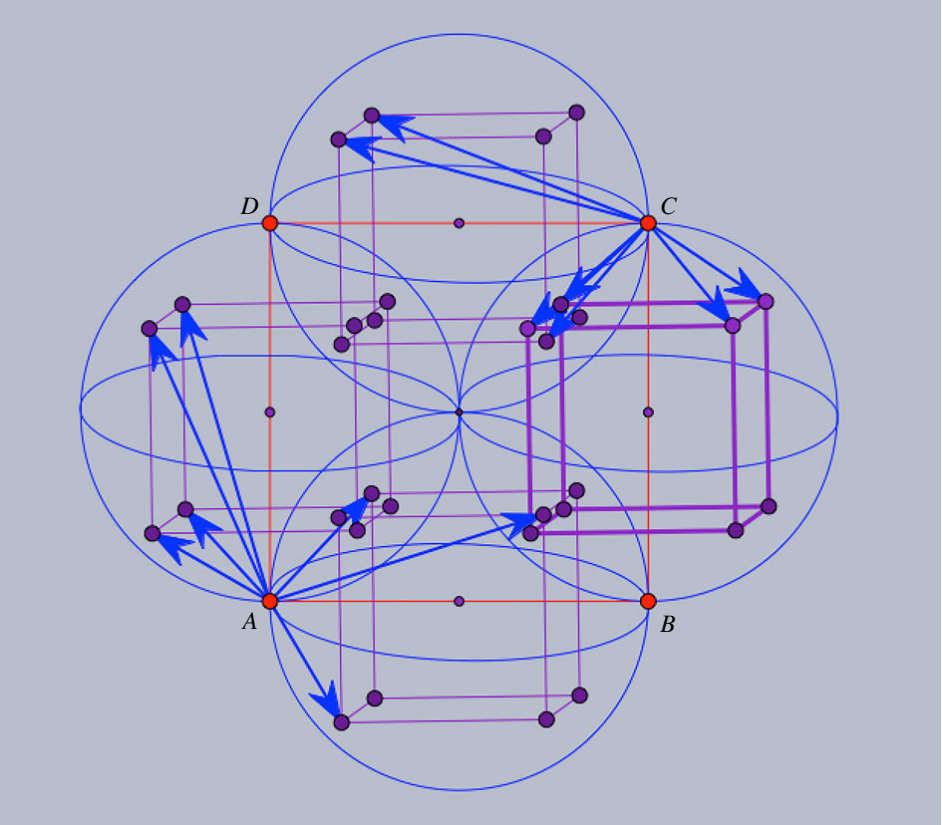
Initial framework blueprint: the 14 representatives for edge orbits are shown as arrows emanating from *A*, respectively *C*. The three edge-vector representatives from *A* to vertex orbit *B* are in the sphere of diameter *AB*. The initial framework is obtained by assembling the edge-vector representative according to their endpoints and periodicity.

For a description of the initial framework, it is enough to indicate 14 edge vectors which provide a complete set of representatives for the periodic graph (G=(V,E),Γ). We use the symbols *A*, *B*, *C*, *D* for the four orbits of vertices V/Γ and refer to figures 1 and 2 for the prescription of initial edge vectors. For instance, the three edge vector representatives from vertex orbit *A* to vertex orbit *B* are shown as arrows emanating from *A* and ending at vertices of the inscribed cube of the sphere with diameter *AB*. Similarly, the four edge vector representatives from vertex orbit *A* to vertex orbit *D* are shown as arrows emanating from *A* and ending at vertices of the inscribed cube of the sphere with diameter *AD*. The remaining seven edge representatives are shown as emanating from *C*, with four arrows in the sphere with diameter *CB* and three arrows in the sphere with diameter *CD*.

## 3. Saturation and symmetry

**Figure 3.**
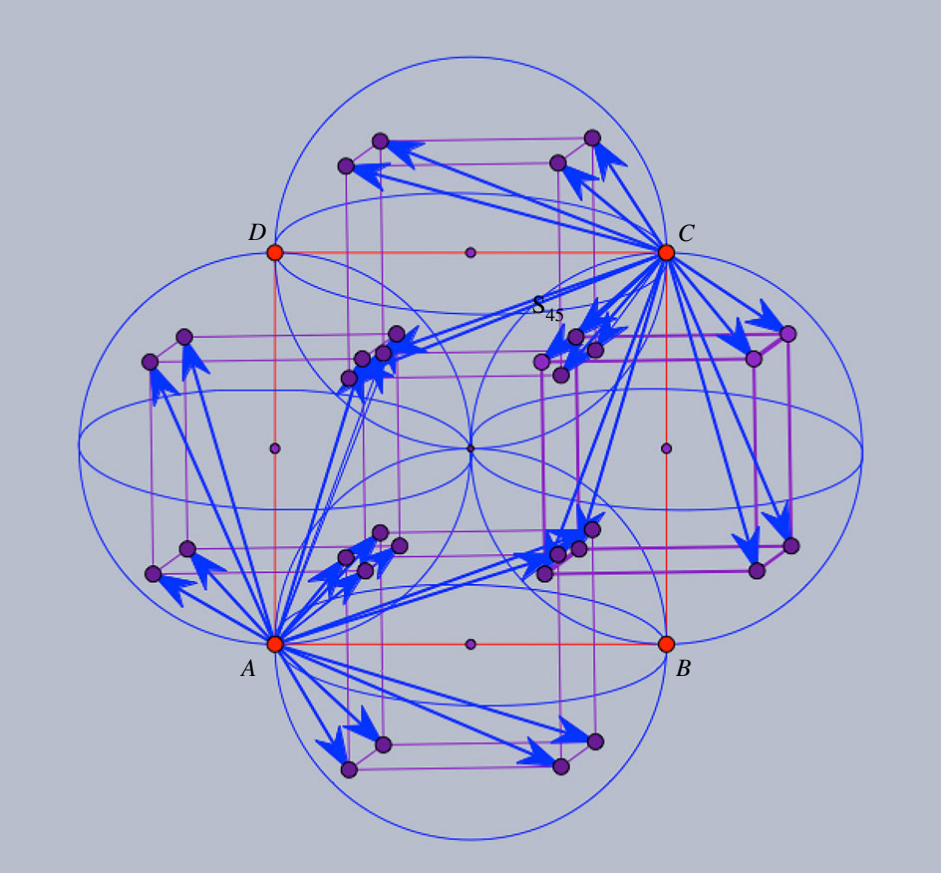
Saturated framework blueprint: there are eight edge vectors from *A* to orbit *B*, ending at the vertices of the inscribed cube of the sphere with diameter *AB* and similarly for *AD*, *CB* and *CD*.

One is tempted to allow more edges in figure 2 and produce a more symmetric design [19]. We will call *saturated* the framework obtained by *adding 18 edge orbits* and resulting in the blueprint diagram shown in figure 3. The diagram already indicates higher crystallographic symmetry for the saturated framework. It will be convenient to make use of the planar configuration obtained by projection along the normal direction to the frontal face of the inscribed cubes.

**Figure 4.**
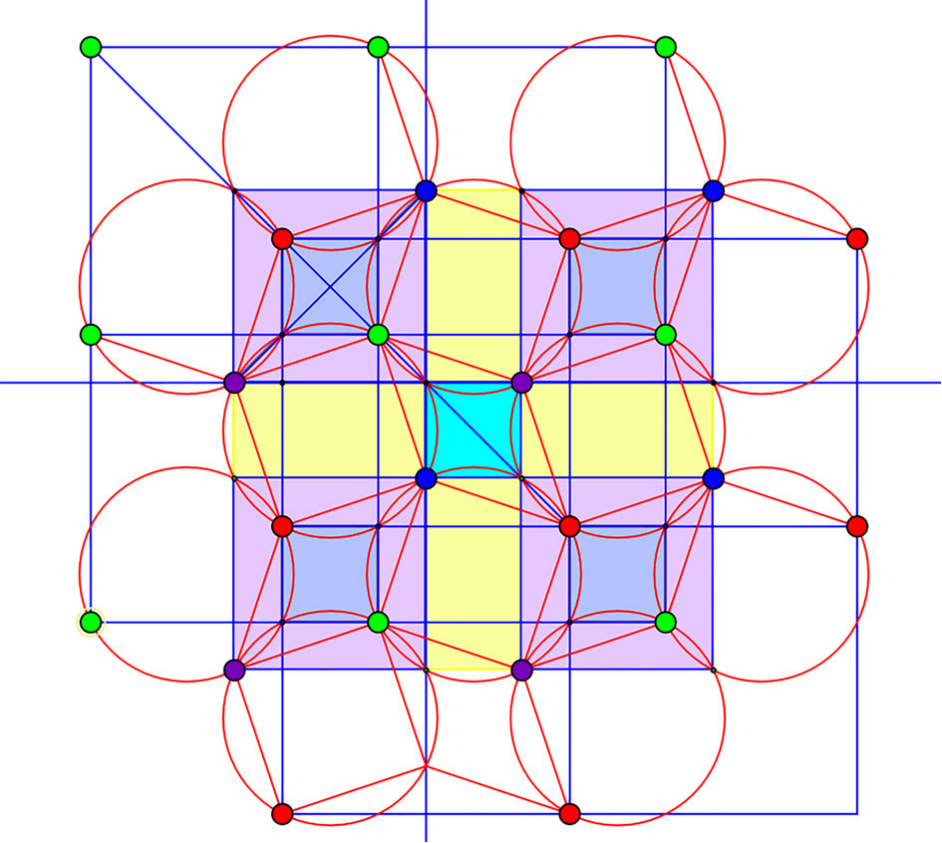
The framework in planar projection.

Thus, in figure 4 projected vertex orbits *A*, *B*, *C*, *D* appear as square lattices (red for *B*, green for *D*). If we view the plane as a slice of the three-dimensional configuration of vertices, we notice that when the slice passes through *B* and *D* vertices, nearby parallel planes with *A* and *C* vertices are half a period below and above the slice. Over the traced circles, one can imagine the corresponding ‘towers’ of vertices and a helix motion around the axis of such a cylindrical tower inducing a crystallographic symmetry permuting cyclically the orbits *A*, *B*, *C*, *D*. Since modulo periodicity, crystallographic symmetries induce automorphisms of the quotient graph, the indicated helical motion corresponds with the rotation of the *ABCD* square in the design diagram. Other symmetries of the diagram (reflection in the *ABCD* plane and reflections exchanging *A* and *C* or *B* and *D*) are also induced by crystallographic symmetries of the framework and will be considered when we present the deformation path of the saturated framework.

## 4. Deformations

In this section, we describe the one-parameter deformation trajectory of the saturated framework, with design diagram shown in figure 3. This trajectory underlies a myriad of local deformations for one-degree-of-freedom periodic frameworks obtained from the saturated one (with 32 edge orbits) by elimination of edge orbits which do not decrease the rank of the rigidity matrix, i.e. maintain rank 14. In particular, one can obtain millions of alternative designs for our initial framework, with blueprint shown in figure 2, by other choices of 14 edge vector representatives, ending at other vertices of the corresponding inscribed cubes.

Our examples show the possibility of *vast numbers* of distinct, non-isomorphic designs which are all kinematically equivalent as a result of their provenance from the common saturated blueprint.

For a simple geometric grasp on the deformation space of the saturated periodic framework, we include some descriptive considerations before obtaining the coordinate formulae.

We fix a vertex representative for orbit *A* (and refer to it simply as vertex *A*). Then, the eight vertices in orbit *B* which are connected to vertex *A* form a cube, with one face closer to *A*, corresponding to short edges, and the opposite face more distant, corresponding to long edges. Likewise, we have eight connected vertices in orbit *D*, forming a translated cube of the orbit *B* connections. By design, there is a unique vertex in orbit *C* (referred to here as vertex *C*), which has its 16 connections to these same cubes in orbits *B* and *D*. The segment *AC* is contained in the mid-plane which cuts through the centres of the *B* and *D* cubes and halves them symmetrically. This plane will be called *the mid-plane* and appears as the projection plane of figure 4.

Reflection in the mid-plane is a crystallographic symmetry of the entire saturated framework. Moreover, the common diagonal plane of the *B* and *D* cubes is orthogonal to the mid-plane and halves the segment *AC* orthogonally. Reflection in this plane is a symmetry of the saturated framework which exchanges *A* and *C* and their respective orbits. A third plane arising from this configuration runs through the segment *AC* and is orthogonal to the other two. Reflection in this plane is a symmetry of the saturated framework which exchanges the vertex orbits *B* and *D*.

If we denote by Σ the group of periodic graph automorphisms generated by the periodicity group Γ and the additional framework symmetries described above, then the quotient group Σ/Γ (which gives the *crystallographic point group* of the framework) has order 16 and is faithfully represented by the induced automorphisms of the quotient of the saturated periodic graph by Γ.

For possible deformations, we look at *A* and the close face of the *B* cube. Since the four edges of this face consist of period vectors, in any periodic deformation, the face remains a parallelogram. Moreover, since *A* remains at the same distance from the vertices of this quadrilateral, the parallelogram must be a rectangle. With similar considerations for the distant face of the *B* cube and then for *C* and its close and distant faces of the same initial *B* cube, we see that the periodicity lattice must deform in such a way that the initial cube remains an orthogonal box, with what we called the frontal face remaining a square. In other words, when we take as periodicity generators the three edge vectors emanating from a vertex of the *B* cube, they are constrained to remain mutually perpendicular, with two of them equal in length. The length of the third generator is determined via the fixed length of the short, respectively long edges from *A* to the evolved *B* box. Finally, this deformation scenario for vertex *A*, box *B* and *C* extends to a one-parameter deformation of the saturated framework which preserves Σ/Γ as the abstract point group.

**Remark.**

In crystallography, a lattice generated by three mutually perpendicular vectors, with two of them of equal length, is called *(primitive) tetragonal* and the one-parameter deformation described here occurs in the *tetragonal crystal system*.

## 5. Explicit formulae

In this section, we present a coordinate description with explicit formulae for the deformation trajectory of the saturated framework. Then, we identify the auxetic interval on this trajectory, using geometrical criteria developed in [2,3].

We start with the design diagram shown in figure 3 and consider the four congruent spheres to have radius 1. Then, the inscribed cubes have edge length $2/\sqrt{3}$. It follows that all *short edges* of the saturated framework have length equal to the distance from *A* to vertices of the close face of cube B (inscribed in the sphere with diameter *AB*), and this length is *s*, with $s^{2}=2-\frac{2}{\sqrt{3}}$. Similarly, all *long edges* of the saturated framework have length equal to the distance from *A* to vertices of the remote face of cube *B*, and this length is ℓ, with $\ell^{2}=2+\frac{2}{\sqrt{3}}$.

For further computations, it will be useful to retain, from the heuristic overview in §4, that the frontal face of the cube *B* deforms as a square, while the four side faces deform as congruent rectangles. We denote by *a* the length of the rectangle edge normal to the frontal face and *b* will stand for the length of the other rectangle edge, which is shared with the frontal face. Thus, the initial cube *B* (which represents a fundamental periodicity domain, commonly called a unit cell) with $a=b=2/\sqrt{3}$, deforms into an orthogonal box with depth *a* and squared frontal face with length and width *b*.

In other words, when following the evolution of the three mutually orthogonal generators of the periodicity lattice, their Gram matrix will be a symmetric 3 × 3 matrix with diagonal entries *α* = *a*^2^, *β* = *b*^2^, *β* = *b*^2^ and zero elsewhere. As emphasized in previous works [2,3,20], the Gram map is a key indicator for periodic deformations in general and particularly relevant for recognizing auxetic behaviour. In the present case, the geometric criterion for auxetic behaviour [2] amounts to: *α* and *β* increase simultaneously (or, in reverse, decrease simultaneously).

We have seen in §4 that *α* and *β* are related. Thus, the *auxetic interval* of the deformation path will be determined from the explicit dependence between them.

**Figure 5.**
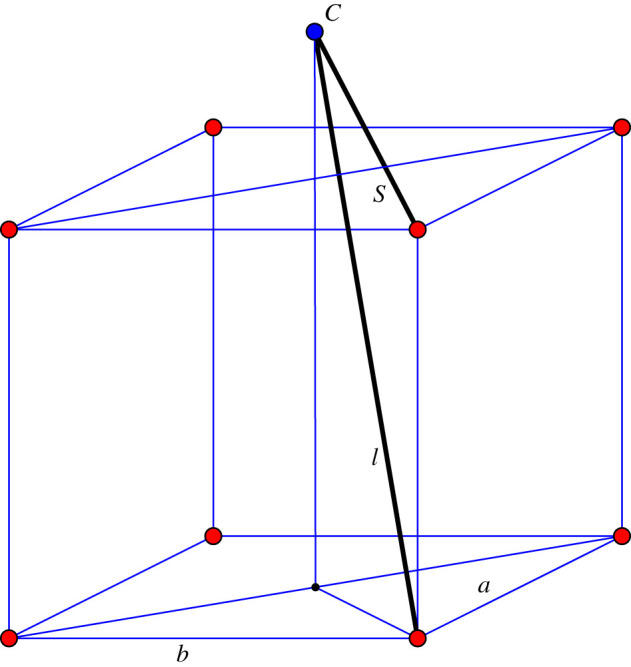
Relation between *a* and *b*.

We find this relation by using figure 5. It shows orthogonal box *B* (with square frontal face) and vertex *C* with a short edge to the close face and a long edge to the remote face. Let *h* denote the length of the perpendicular from *C* to the remote face. Then, we have:

$$a^{2}+b^{2}=4(\ell^{2}-h^{2})\;\text{and}\;{\left(h-b\right)}^{2}+(\ell^{2}-h^{2})=s^{2}.$$

With *α* = *a*^2^ and *β* = *b*^2^, elimination of *h* from these two equations and the values for *s*^2^ and ℓ^2^ found above, result in the quadratic relation:

5.1 $$2\beta^{2}+\alpha \beta -8\beta +\frac{16}{3}=0.$$

This equation represents a hyperbola in the affine space with coordinates (*α*, *β*) and the one-dimensional deformation space of the saturated framework can be represented by a certain arc of the hyperbola which contains the initial configuration with (*α*, *β*) = (4/3, 4/3). The extent of the arc and various points corresponding to significant configurations will be detected below. We can use *β* as *parameter for the deformation* and then *α*, as a function of *β*, is given by

5.2 $$\alpha =\alpha (\beta )=8-2\beta -\frac{16}{3}\beta^{-1}.$$

### 5.1. Deformation interval

We determine first the interval for the geometric deformation of the saturated framework and note that configurations with self-crossing are allowed. Since *α*, *β* > 0, we must have

5.3 $$8\beta -2\beta^{2}-\frac{16}{3}>0,$$

and *β* must be between the roots of the quadratic polynomial in (5.3), that is:

5.4 $$\beta \in \left(2-\frac{2}{\sqrt{3}},2+\frac{2}{\sqrt{3}}\right)=(s^{2},\ell^{2}).$$

At the endpoints of this interval *α* = 0 and three-dimensional periodicity breaks down.

### 5.2. Auxetic interval

When allowing self-crossing, the only condition for *auxetic behaviour* of the saturated framework is that *α* increases when *β* increases in the deformation interval (5.4) [2]. Thus, we look at the derivative of the function *α*(*β*) obtained above in (5.2). The critical points satisfy the equation

5.5 $$\alpha^{\prime }(\beta )=-2+\frac{16}{3}\beta^{-2}=0,$$

and we have just $\beta =2\sqrt{2}/\sqrt{3}$ in the deformation interval, corresponding with a maximum of $\alpha (2\sqrt{2}/\sqrt{3})=8(1-\sqrt{2/3})$ there. Thus, the *auxetic interval* is

5.6 $$\beta \in \left(2-\frac{2}{\sqrt{3}},2\sqrt{\frac{2}{3}}\right)$$

### 5.3. Parametrized deformation path

Our parameter is $\beta \in (2-\frac{2}{\sqrt{3}},2+\frac{2}{\sqrt{3}})$, with initial configuration obtained for *β* = 4/3.

The *periodicity lattice generators* are along the standard basis *e*_1_, *e*_2_, *e*_3_, namely:

5.7 $$\begin{array}{rl} & \lambda_{1}=\beta^{1/2}\cdot e_{1}\\ & \lambda_{2}=\beta^{1/2}\cdot e_{2}\\ \text{and}\;\;\;\;\;\; & \lambda_{3}=\alpha^{1/2}e_{3}={\left(8-2\beta -\frac{16}{3\beta }\right)}^{1/2}\;\cdot \;e_{3}. \end{array}\}$$

The *origin of the Cartesian axes* is kept at the midpoint of *AC*. Since |*AC*|^2^ = 2(*h* − *b*/2)^2^, we find that

5.8 $$A=-{\left(3\beta \right)}^{-1/2}(e_{1}+e_{2})\;\text{and}\;C=-A$$

The centre of the *B* box is at

$$c(B)=-{\left(3\beta \right)}^{-1/2}(e_{1}-e_{2}).$$

The *B* vertex is at

5.9 $$B=c(B)-\frac{1}{2}(\lambda_{1}+\lambda_{2}+\lambda_{3}),$$

and the remaining vertices of the *B* box are:

$$B+\lambda_{1},\;B+\lambda_{2}\;\text{and}\;B+\lambda_{3}$$

and

$$B+\lambda_{2}+\lambda_{3},\;B+\lambda_{3}+\lambda_{1},\;B+\lambda_{2}+\lambda_{2}\;\text{and}\;B+(\lambda_{1}+\lambda_{2}+\lambda_{3}).$$

The centre of the *D* box is at

$$c(D)=-c(B)={\left(3\beta \right)}^{-1/2}(e_{1}-e_{2}).$$

The *D* vertex is at

5.10 $$D=-B=c(D)+\frac{1}{2}(\lambda_{1}+\lambda_{2}+\lambda_{3})$$

and the remaining vertices of the *D* box are:

$$D-\lambda_{1},\;D-\lambda_{2}\;\text{and}\;D-\lambda_{3}$$

and

$$D-\lambda_{2}-\lambda_{3},\;D-\lambda_{3}-\lambda_{1},\;D-\lambda_{1}-\lambda_{2}\;\text{and}\;D-(\lambda_{1}+\lambda_{2}+\lambda_{3}).$$

The *central symmetry in the origin* exchanges *A* with *C* and *B* with *D*.

**Figure 6.**
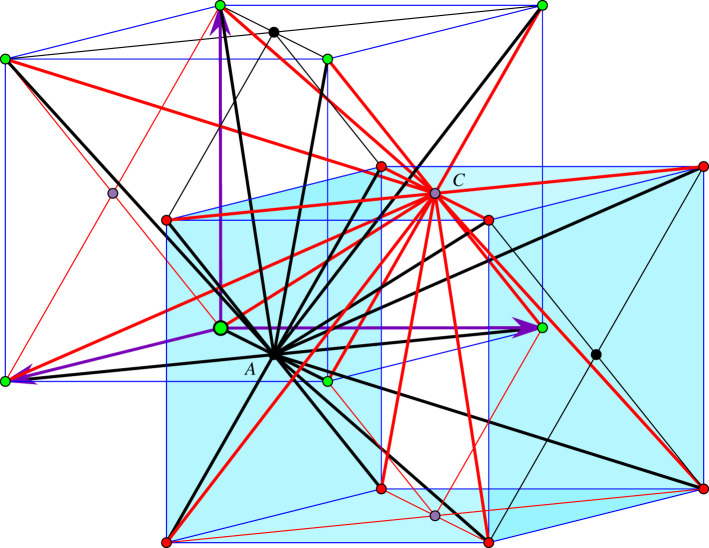
The saturated configuration corresponding to $\beta =4/\sqrt{3}$. Vertex *A* has reached a rectangular face of box *B* as well as a rectangular face of box *D*. There are 16 edges emanating from *A* (shown in black) and 16 edges emanating from *C* (shown in red). The generators of the periodicity lattice are shown as arrows.

We illustrate in figure 6 the essentials of the configuration obtained for $\beta =4/\sqrt{3}$. This case, although outside the auxetic interval, is more convenient for visualizing, since *A* is now exactly at the centre of a rectangular face of box *B* and at the centre of a rectangular face of box *D*.

## 6. Self-crossing

For three-dimensional printing of samples, one has to investigate the problem of self-crossing, since periodic framework configurations may have intersecting edges. Two intersecting edges imply a coplanarity condition for their endpoints.

For the saturated framework under examination, projection on a mid-plane, as described above and illustrated in figure 4 will serve finding self-crossings by elementary geometrical considerations.

First, we observe that, by symmetry, any self-crossing will be manifest on the edges from vertex *A* to box *B*. Actually, by reflection in the mid-plane, the necessary inspection is reduced to the four edges from *A* to what we called the ‘frontal face’ of the *B* box.

**Figure 7.**
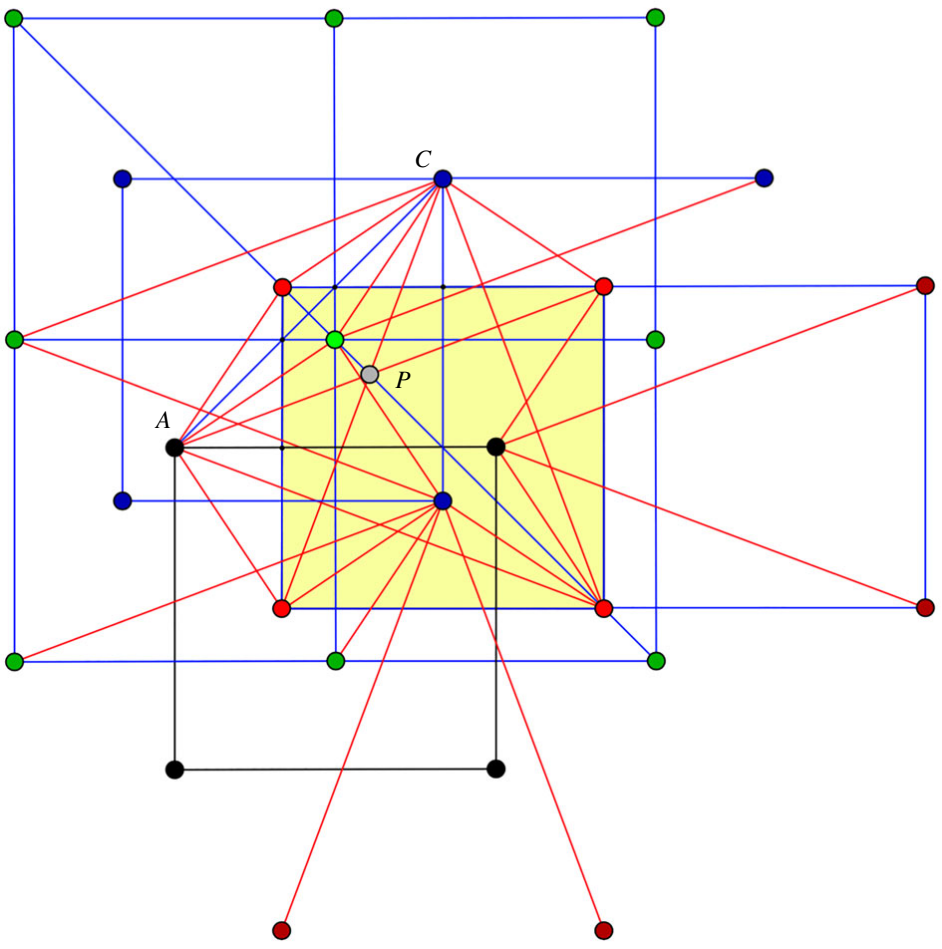
Mid-plane projection for exposing intersections between edges of type *AB* and edges of type *CB* or *CD*. Point *P* marks the projection of intersecting long edges of type *AB* and *CB*.

In figure 7, adapted for self-crossing detection, projections of edges are shown in red. The relevant vertices in the mid-plane belong to orbits *A* (black) and *C* (blue), while relevant vertices from orbit *B* (red) and *C* (green) should be imagined in space in the nearby parallel plane. Edges to be inspected for possible intersection have one vertex in the mid-plane and the other vertex in the parallel plane above. The highlighted square corresponds with the projection of the *B* box (which represents the unit cell of the framework). The detailed inspection of this figure allows the identification of possible intersections between an *AB* edge and a *CB* or *CD* edge. For a generic configuration along the deformation path, this kind of inspection finds possible only a crossing of type *AB* with type *CB*. In projection, the common point is marked as *P*. A unit cell has two intersections of this type, symmetric by mid-plane reflection. The visual confirmation amounts to observing that the segment AC is parallel with the segment between the other two endpoints of the implicated edges.

**Figure 8.**
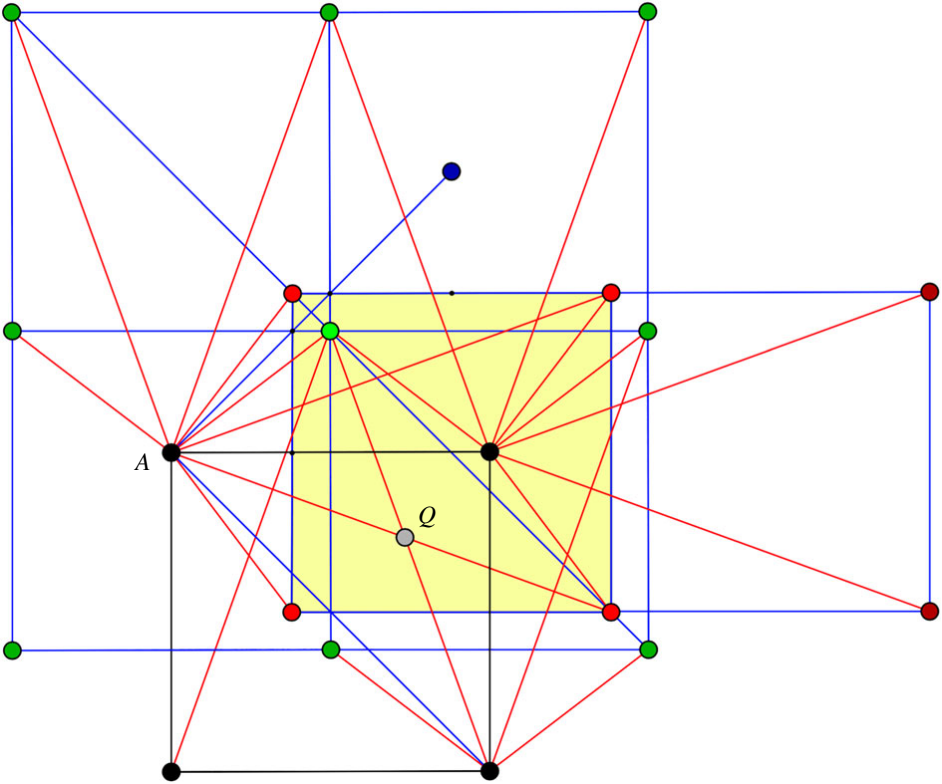
Mid-plane projection for exposing intersections between edges of type *AB* and edges of type *AB* or *AD*. Point *Q* marks the projection of intersecting long edges of type *AB* and *AD*.

A similar examination, based on figure 8, can be conducted for possible intersections between an *AB* edge and some other edge of type *AB* or *AD*. For a generic configuration along the deformation path, one finds intersections of type *AB* with type *AD* projecting to the point *Q*. Again, a unit cell contains two intersections of this type, symmetric by mid-plane reflection.

We conclude in this manner that, at any point of the deformation path, the corresponding saturated configuration has, modulo periodicity, four pairs of intersections between long edges of the following type:

AB∩CB,AB∩AD,AD∩CDandCB∩CD.

**Figure 9.**
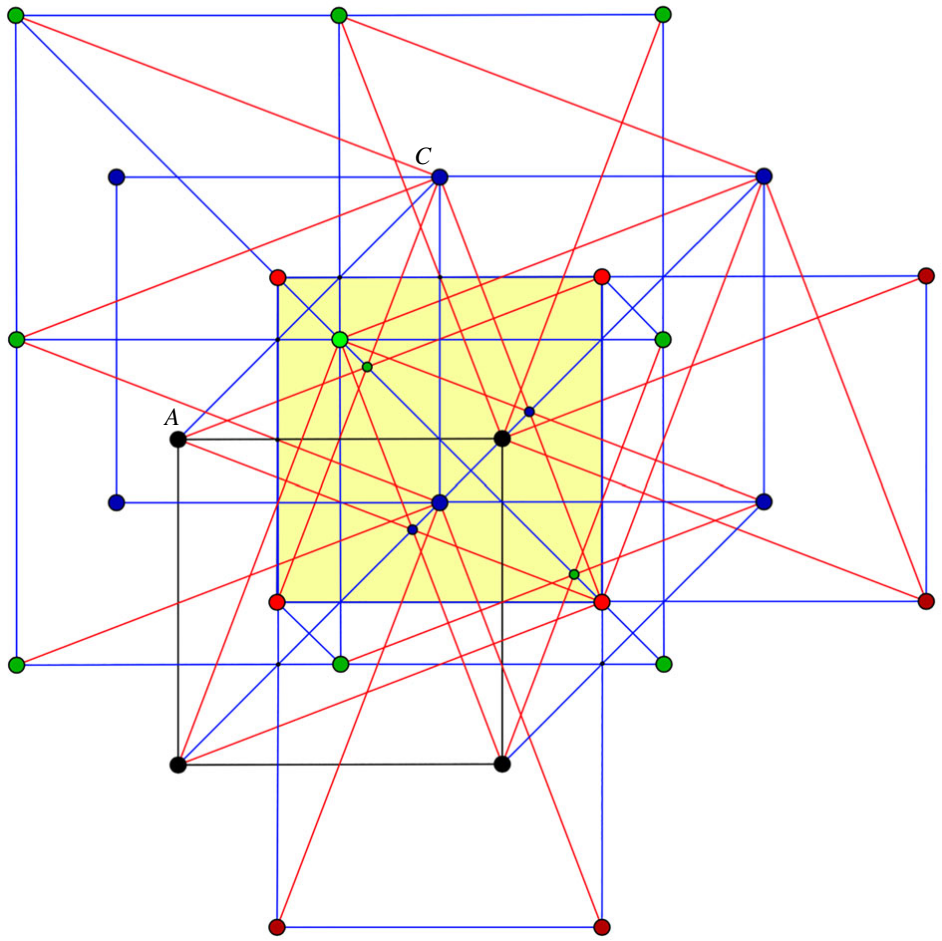
Mid-plane projection showing long edge self-crossings.

These pairs result from reflection in a mid-plane (which preserves the vertex orbits *A*, *B*, *C*, *D*), while the four types of self-crossing result from crystallographic symmetries with non-trivial action on vertex orbits. In figure 9, each pair project to a single point. The above four types of intersection are indicated in a highlighted projection of a B box by points of smaller size than vertices.

At isolated points of the deformation path, one may encounter new types of self-crossing and we look for the nearest configurations of the initial saturated framework where additional self-intersections occur.

**Figure 10.**
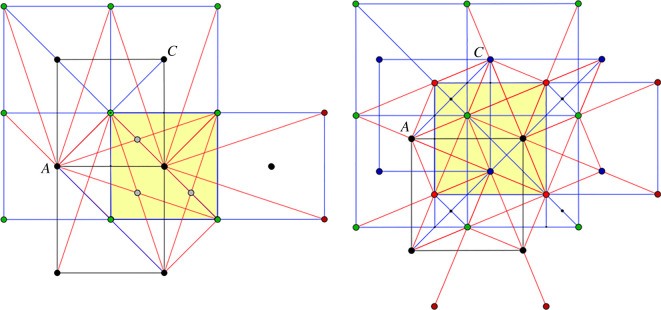
Mid-plane projections at the endpoints of the inner deformation interval, when additional self-intersections occur.

The configurations of interest are shown in figure 10, by way of mid-plane projection. On the left, vertex orbits *B* and *D* have coalesced and vertex orbits *A* and *C* likewise, resulting in superimposed edges. On the right, we will presently recognize the configuration corresponding to the end of the auxetic interval. Since collinearity in projection implies coplanarity in space, we see that, in both cases, the vertices can be conceived as disposed in parallel planes which contain representatives from all four orbits *A*, *B*, *C*, *D*.

The main reason for finding this restricted deformation window, called the *inner deformation interval*, is that by adequate removal of eight edge orbits, the initial saturated framework (with 32 edge orbits) turns into a framework (with 24 edge orbits) without self-crossing along the interval.

For the computation of the parameter *β* at the endpoints of the inner deformation interval, we use the *squared distance* between the vertices marked *A* and *C*, namely:

6.1 $$|AC{|}^{2}=\frac{8}{3\beta }.$$

We note that, when vertex orbits *B* and *D* coincide, although vertex orbits *A* and *C* coincide as well, the vertex representatives marked *A* and *C* are not superimposed. Actually, we have |*AC*|^2^ = 2*β*, resulting in $\beta =2/\sqrt{3}$. The other configuration has |*AC*|^2^ = *β*, resulting in $\beta =2\sqrt{2}/\sqrt{3}$, as found earlier in (5.6) for the end of the auxetic interval.

The *inner deformation interval* is given by

6.2 $$\beta \in \left(\frac{2}{\sqrt{3}},\frac{2\sqrt{2}}{\sqrt{3}}\right).$$

## 7. Rank considerations

Based on the parametrized deformation path given above in formulae (5.7) to (5.10), we can write down the *rigidity matrix* at any point of the path [1].

We put:

e=AB=B−A and f=AD=D−A,hence CD=−e and CB=−f.

With this notation, the eight edge vectors from vertex *A* to orbit *B* are:

$$e,\;e+\lambda_{1},\;e+\lambda_{2}\;\text{and}\;e+\lambda_{3}$$

and

$$e+\lambda_{2}+\lambda_{3},\;e+\lambda_{3}+\lambda_{1},\;e+\lambda_{1}+\lambda_{2}\;\text{and}\;e+\lambda_{1}+\lambda_{2}+\lambda_{3}$$

The eight edge vectors from vertex *A* to orbit *D* are:

$$f,\;f-\lambda_{1},\;f-\lambda_{2}\;\text{and}\;f-\lambda_{3}$$

and

$$f-\lambda_{2}-\lambda_{3},\;f-\lambda_{3}-\lambda_{1},\;f-\lambda_{1}-\lambda_{2}\;\text{and}\;f-\lambda_{1}-\lambda_{2}-\lambda_{3}.$$

With similar listing of the edge vectors from vertex *C* to orbit *D* and orbit *B*, the 32 × 21 rigidity matrix, with edge vectors treated as *row vectors* and rows ordered as type *AB*, *AD*, *CD* and *CB*, takes the form:

7.1 $$\left(\begin{array}{ccccccc} -e & e & 0 & 0 & 0 & 0 & 0\\ -e-\lambda_{1} & e+\lambda_{1} & 0 & 0 & e+\lambda_{1} & 0 & 0\\ -e-\lambda_{2} & e+\lambda_{2} & 0 & 0 & 0 & e+\lambda_{2} & 0\\ -e-\lambda_{3} & e+\lambda_{3} & 0 & 0 & 0 & 0 & e+\lambda_{3}\\ \cdots & \cdots & \cdots & \cdots & \cdots & \cdots & \cdots \\ -f & 0 & 0 & f & 0 & 0 & 0\\ -f+\lambda_{1} & 0 & 0 & f-\lambda_{1} & -f+\lambda_{1} & 0 & 0\\ \cdots & \cdots & \cdots & \cdots & \cdots & \cdots & \cdots \\ 0 & 0 & e & -e & 0 & 0 & 0\\ 0 & 0 & e+\lambda_{1} & -e-\lambda_{1} & e+\lambda_{1} & 0 & 0\\ \cdots & \cdots & \cdots & \cdots & \cdots & \cdots & \cdots \\ 0 & -f & f & 0 & 0 & 0 & 0\\ 0 & -f+\lambda_{1} & f-\lambda_{1} & 0 & -f+\lambda_{1} & 0 & 0\\ \cdots & \cdots & \cdots & \cdots & \cdots & \cdots & \cdots \end{array}\right).$$

The initial saturated framework has *β* = 4/3 and all entries in (7.1) belong to the quadratic extension $Q(\sqrt{3})$ of the rational field Q. Thus, rank computations can be carried out with absolute precision and we simply state the results.

First of all, the rank of the rigidity matrix is 14, and the 14 rows of the initial framework are independent. This means (via the implicit function theorem) that, as intended, the initial framework has a smooth local deformation space of dimension one (i.e. has one degree of freedom) and the saturated framework has the same local deformation space.

Let us recall from [1] that, for any given framework, a *periodic self-stress* or simply self-stress, is a linear dependence between the rows of the rigidity matrix. If we denote by *d* the dimension, *n* the number of vertex orbits, *m* the number of edge orbits, *r* the rank of the rigidity matrix, *f* the dimension of the space of infinitesimal flexes (i.e. the dimension of the tangent space to the deformation space at the given framework) and by *s* the dimension of the space of self-stresses, we have the following basic relations:

(i) the rigidity matrix has *m* rows and *nd* + *d*^2^ columns;
(ii) $r+f=nd+\left(\begin{array}{c} d\\ 2 \end{array}\right)$;
(iii) *r* + *s* = *m*.

For our initial saturated framework, we have:

d=3,n=4,m=32,r=14,f=1ands=18,

satisfying the required relations. Thus, each edge orbit added to the initial framework increased the dimension of the space of self-stresses by one and the resulting *s* = 18 is fairly high.

At this point, we may consider *removing edge orbits* from the saturated framework and regaining a framework structure with one degree of freedom and 14 edge orbits. All these structures have the same kinematics (and the same behaviour of the periodicity lattice) as the initial framework, which is one of them. Thus, all represent alternative *auxetic designs* and we call them *cognate frameworks*.

The relevant combinatorial notion governing the removal possibilities is that of *matroid basis polytope* [21–23]. Indeed, we have to remove 18 rows from the rigidity matrix and remain with 14 independent rows, i.e. a basis in the linear matroid defined by the rigidity matrix. The vector in {0, 1}^32^ with 0 for the removed rows and 1 for the remaining rows defines a vertex of the matroid basis polytope, which is the convex hull of all such vertices in the unit cube [0, 1]^32^. We do not undertake here a more detailed investigation of the resulting polytope. Preliminary computations produced millions of vertices. Using a formula established in [23], it can be shown that the polytope has full dimension 31.

In the following section, we discuss a saturation scenario in dimension two, which is less involved computationally and refers to some well-known designs.

## 8. A planar case

We consider a periodic framework in dimension two with *n* = 4 vertex orbits and *m* = 8 edge orbits. The framework has 1 d.f. and allows a saturation with 12 edge orbits.

**Figure 11.**
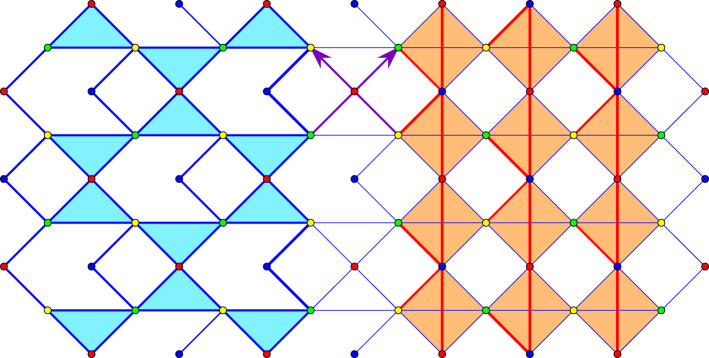
Initial framework (left) and saturation (right) with four additional edge orbits, marked in red. Two generators of the periodicity lattice are shown as arrows.

The initial configuration and its saturation are displayed in figure 11, which emphasizes that the initial structure consists of a Kagome framework with a fourth vertex orbit connected by two edge orbits, while saturation, with four additional edge orbits (in red), produces rigid squares with the ‘rotating squares’ framework kinematics [24–26].

**Figure 12.**
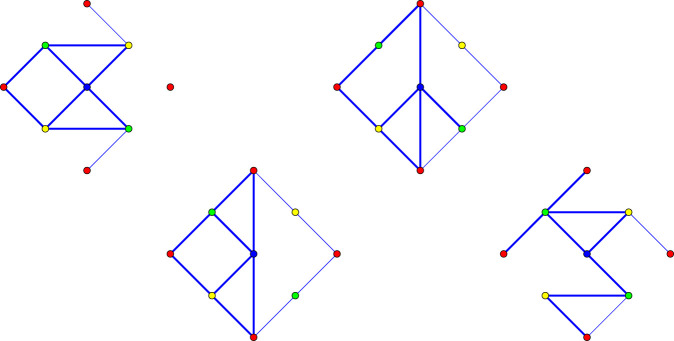
Unit cells of four cognates. The eight edge representatives are thick segments. Thinner segments are periodic repeats on the boundary of the cell.

All vertex coordinates are integers and the rigidity matrix for the saturated framework is 12 × 12, with rank 8. Thus, the vertices of the matroid basis polytope can be computed by elementary routines. The result is a polytope of dimension 11, with 282 vertices. In figure 12, we show the unit cell of four cognate frameworks, with the initial structure on the left.

## 9. Conclusion

The present study highlights two aspects of geometric auxetics: first, that the general methodology for *auxetic periodic design*, established in [3], can adequately address various specifications and second, that, as in the classical theory of finite linkages, there is a notion of *cognate periodic frameworks* based on a shared local deformation space.

The phenomenon of cognates is explained by a process of *saturation*, permitted by certain flexible bar-and-joint structures, which allow particular additions of bars without altering their local kinematic behaviour. In the periodic context, we require the initial framework to have a smooth local deformation space and no self-stress. Then, a bar orbit can be added when it produces a self-stress in the new structure. Cognates are obtained by reversing a saturation, that is, by eliminating the added self-stress through a sequence of bar orbit removals. We have shown that the possible reversals correspond to the vertices of a *matroid basis polytope* (defined by the rigidity matrix of the saturated framework).

It is apparent from our examples that saturation and reversal may result in *large numbers of cognates*, even after equivalence under symmetries. For auxetic design (and especially one degree of freedom auxetic design), this is quite relevant, since unexpected possibilities and relations are brought to light.

## Data Availability

This article has no additional data.
